# “I pick you”: the impact of fairness and race on infants’ selection of social partners

**DOI:** 10.3389/fpsyg.2014.00093

**Published:** 2014-02-12

**Authors:** Monica P. Burns, Jessica A. Sommerville

**Affiliations:** ^1^Department of Psychology, University of WashingtonSeattle, WA, USA; ^2^Center for Child and Family Well-being, Department of Psychology, University of WashingtonSeattle, WA, USA

**Keywords:** fairness, race, social partners, social selections, resource distribution

## Abstract

By 15 months of age infants are sensitive to violations of fairness norms as assessed via their enhanced visual attention to unfair versus fair outcomes in violation-of-expectation paradigms. The current study investigated whether 15-month-old infants select social partners on the basis of prior fair versus unfair behavior, and whether infants integrate social selections on the basis of fairness with the race of the distributors and recipients involved in the exchange. Experiment 1 demonstrated that after witnessing one adult distribute toys to two recipients fairly (2:2 distribution), and another adult distribute toys to two recipients unfairly (1:3 distribution), Caucasian infants selected fair over unfair distributors when both distributors were Caucasian; however, this preference was not present when the fair actor was Asian and the unfair actor was Caucasian. In Experiment 2, when fairness, the race of the distributor, and the race of the recipients were fully crossed, Caucasian infants’ social selections varied as a function of the race of the recipient advantaged by the unfair distributor. Specifically, infants were more likely to select the fair distributor when the unfair recipient advantaged the Asian (versus the Caucasian) recipient. These findings provide evidence that infants select social partners on the basis of prior fair behavior and that infants also take into account the race of distributors and recipients when making their social selections.

## INTRODUCTION

The ability to actively select social partners on the basis of relevant characteristics critically shapes the acquisition of knowledge; selecting social partners constrains the kinds of people to which an individual is exposed which can, in turn, guide subsequent attitudes and behaviors. A variety of research suggests that adults systematically select social partners on the basis of several dimensions, including an individual’s social history and an individual’s social category membership. For example, adults prefer individuals who are more generous toward others in economic games (Page et al., 2005), and they tend to affiliate with social partners who are similar to themselves in terms of race, age, and socioeconomic status (McPherson et al., 2001). The roots of the tendency to select social partners on the basis of social history and social category membership can be traced back to childhood: children tend to have friends who are of the same gender (Martin and Fabes, 2001) and of the same race (Katz, 2003). In experimental paradigms, children preferentially select individuals who share characteristics with the self (Shutts et al., 2013) and who previously acted cooperatively over uncooperative individuals (Dunfield et al., 2013).

A critical, unanswered question is whether infants also make systematic social selections on the basis of relevant social dimensions. Emerging evidence suggests that infants may consider social history when selecting between two agents; when given the choice between a prosocial puppet who previously helped another puppet retrieve a toy and an antisocial puppet who previously prevented the puppet from retrieving a toy, 5-month-old infants pick the prosocial puppet (Hamlin and Wynn, 2011). Moreover, when given the opportunity to select toys associated with native language speakers over those associated with non-native language speakers (i.e., those who speak with an accent) infants prefer toys offered by native language speakers, suggesting that infants may use social category information to guide their social selections (Kinzler et al., 2012).

The goal of the current study is to investigate infants’ ability to select social partners on the basis of social history, and to investigate whether and how infants’ social selections are altered when social history information conflicts with social category information. In the present study, we operationalized social history in terms of whether an actor had previously distributed toys equally or unequally to recipients. Past work with adults suggests that a “principle of equality” (Deutsch, 1975) guides adults’ social judgments and evaluations: that, all other things considered, goods should be divided equally to recipients. In the context of economic games, adults divide resources between oneself and an anonymous social partner equally (Fehr and Fischbacher, 2003; Henrich et al., 2005) and punish individuals who do not do so and seek redistribute goods equally (Dawes et al., 2007; Johnson et al., 2009). Recent studies suggest that infants appear to be sensitive to fairness violations by 15 months of age or earlier: after watching an individual distribute crackers to two recipients in a violation-of-expectancy paradigm, infants show enhanced attention to an unfair outcome (i.e., 1:3 distribution) versus a fair outcome (2:2 distribution) suggesting they expect goods to be distributed equally (Schmidt and Sommerville, 2011; Sommerville et al., 2013; see also Geraci and Surian, 2011; Sloane et al., 2012).

The novel question addressed in this study was whether infants could use prior information about an individual’s fair versus unfair behavior to guide their own selection of social partners. If so, these findings would add to the current literature by demonstrating that infants are not only aware of fairness norms as reflected by their expectations of third-party interactions, but also that infants use their awareness of such norms to guide their social behavior.

In addition to asking whether infants consider an individual’s prior history of fair and unfair behavior in making their social selections, we asked whether information about the social category membership of an individual affects infants’ social selections. In the current study, we operationalized social category membership in terms of the race of the individuals, as adults systematically use race as an indicator of social category membership (Fiske and Neuberg, 1990; Hewstone et al., 1991; Stangor et al., 1992). Evidence suggests that same-race social preferences are in place by the school-aged years: elementary-aged children reveal a racial bias in their friendships and in peer nominations, preferring same-race peers (Aboud et al., 2003; Bellmore et al., 2007). Work using experimental paradigms also demonstrates that the impact of race on children’s social preferences can be traced back to at least the early preschool years. Three- to five-year-old children systematically select same-race unfamiliar peers and adults as potential friends over those of another race (Katz and Kofkin, 1997; Kinzler and Spelke, 2011). Moreover, children prefer others who exclusively affiliate with members of their ingroup: Caucasian preschoolers selectively preferred characters in vignettes who were depicted playing with other Caucasian characters as potential friends, rather than those depicted with Black characters (Castelli et al., 2007). In addition to possessing race-based social preferences children as young as three also show adult-like implicit race biases in an age-appropriate version of the Implicit Association Task (Dunham et al., 2013).

We were motivated to investigate the impact of race on infants’ social selections as current research suggests infants show an early sensitivity to race in their attentional patterns. Evidence from visual preference studies suggests that race influences infants’ looking preferences for different faces: infants as young as 3 months of age prefer to look at same-race over other-race faces (Kelly et al., 2005). Existing research on social selections based on race in infancy, however, has yielded mixed results. On the one hand, preliminary findings using live, interactive paradigms with 12-month-old infants indicate that Caucasian infants prefer to take toys offered by Caucasian versus Asian individuals when given no other information about the individuals (Shin et al., 2011). On the other hand, Kinzler and Spelke (2011) found that 10-month-old infants selected toys associated with a Caucasian adult at equal rates as toys associated with a Black adult, providing no evidence for race-based social selections in infancy. Thus, the extent to which infants consider race in their social selections is an open question.

In advance of prior work, the current study sought to investigate whether and how infants integrate multiple dimensions – fairness and race – in making their social selections. In the real world, individuals often select social partners under conditions in which different social dimensions are either in conflict or conflated. Thus, we investigated whether and how infants integrate different dimensions of social information into their social decision-making processes. Experiment 1 investigated whether infants would select individuals on the basis of previously fair behavior when the race of the two individuals was controlled for (i.e., both were Caucasian), and when the fair individual was of a different race than the unfair actor and the infant (i.e., the fair actor was Asian and the unfair actor was Caucasian). These manipulations allowed us to ask whether infants had a baseline preference for fair over unfair individuals, and how and whether this preference was affected by the race of the distributors.

In Experiment 2, we fully crossed fairness with the race of both the distributors and the recipients. Thus, in this study infants had the opportunity to select distributors on the basis of prior fair or unfair behavior, on the basis of race, or on the basis of the consequences of the distributors’ actions for their own or other race members. Critically, this experiment allowed us to assess how infants make social selections when faced with competing motivations: concerns about adherence to socio-moral norms, motivations to interact with individuals of the same social category, and considerations of the outcomes of distributive actions for self and same-race members. Past research suggests that each of these factors not only independently affect adult’s and children’s behavior and social selections, but can also interact in interesting ways to impact social preferences. For example, adults preferred a person who distributed difficult tasks within a group fairly compared to one who distributed such tasks unfairly. However, this preference for the fair distributor was diminished when the unfair distributor divided the tasks in a way that disadvantaged an outgroup member. Under those circumstances, adults endorsed the fair and unfair distributors at equal rates, suggesting adults’ evaluations of the distributors were affected by competing considerations, namely, the fairness of the distributor and the impact of his distribution on outgroup members (Platow et al., 1997).

Taken together, the results of Experiment 1 and 2 inform whether infants use prior fair and unfair behavior to guide their social selections and how and whether this information is used to guide social selections in the face of competing motivations.

## EXPERIMENT 1

In Experiment 1, we investigated infants’ social selections of fair versus unfair distributors, and whether such selections varied as a function of the race of the distributors. In one condition, both distributors were Caucasian and both recipients were Asian. This context provides a particularly stringent test of infants’ fairness concerns. First, examining infants’ social selections after observing third-party interactions circumvents reward history issues that can arise in the context of first-person interactions. Second, other scholars have argued that the expectation that norms extend across social categories is a hallmark of moral principles (versus social conventions; see Turiel, 1983). Prior work demonstrates that infants of this age show sensitivity to violations to fairness norms in their visual responses, and that such sensitivity relates to infants’ prosocial behavior (e.g., Schmidt and Sommerville, 2011; Sommerville et al., 2013), suggesting that infants may view fair distributions of goods as not only conforming to a social convention but also to a moral rule or principle. Thus, we expected infants to systematically select the fair actor in this condition.

In a second condition, we pitted fairness against race by investigating infants’ selections of a fair Asian distributor versus an unfair Caucasian distributor. Past work using similar paradigms provides evidence that Caucasian infants have a baseline preference for Caucasian over Asian individuals in the absence of any other information about these individuals (Shin et al., 2011). In this condition, there are three potential outcomes. First, infants may make social selections strictly on the basis of the distributors’ previously fair (versus unfair) behavior and ignore race, systematically selecting the fair actor. Second, infants may make selections strictly on the basis of race, in which case we predicted that infants would select the unfair Caucasian distributor over the fair Asian distributor, given prior work suggesting that Caucasian infants prefer Caucasian to Asian faces (Kelly et al., 2005) and Caucasian to Asian individuals (Shin et al., 2011). Third, these pieces of information may compete with one another, in which case infants may select the Asian fair distributor (versus the Caucasian unfair distributor) at rates roughly equivalent to chance.

Distributors in the current study were Caucasian or Asian. In order to make same- versus other- race comparisons, and because the population from which our sample was drawn was predominantly Caucasian, we limited our sample to Caucasian infants.

### METHODS

#### Participants

Forty 15-month-old Caucasian infants participated in Experiment 1 (22 females; mean age = 15 months, 12 days, range = 14 months, 26 days to 16 months, 2 days). Infants were randomly assigned to the Caucasian Fair/Caucasian Unfair condition (henceforth CF/CUF; *n* = 20, 10 females, mean age = 15 months, 10 days) or the Asian Fair/Caucasian Unfair condition (henceforth AF/CUF; *n *= 20, 12 females, mean age = 15 months, 13 days). All infants were full-term and typically developing. Participants were recruited from a database of parents who had volunteered to participate in experimental studies. Data from 18 additional infants were excluded because of failure to respond at all during the choice trials (*n = *5 in the CF/CUF condition, *n* = 9 in the AF/CUF), fussiness (*n = *1 in the CF/CUF condition, *n* = 2 in the AF/CUF), or due to procedural errors (*n* = 1 in the CF/CUF condition).

#### Procedure

The infant viewed a distribution phase followed by three choice trials^1^.

***Distribution***. The infant watched a live distribution that involved four actors: two distributors (both Caucasian for the CF/CUF condition; one Caucasian and one Asian for the AF/CUF condition) and two recipients (both Asian)^2^. **Table 1** describes the respective role as of the distributor and recipient as a function of fairness and race for both Experiments 1 and 2.

**Table 1:** Race of distributors and recipients as a function of condition and experiment.

Condition (Experiment)	Fair distributor	Unfair distributor	Advantaged recipient	Disadvantaged recipient
CF/CUF (Experiment 1)	Caucasian	Caucasian	Asian	Asian
AF/CUF (Experiment 1)	Asian	Caucasian	Asian	Asian
CF/AUF: CR+ (Experiment 2)	Caucasian	Asian	Caucasian	Asian
CF/AUF: AR+ (Experiment 2)	Caucasian	Asian	Asian	Caucasian
AF/CUF: CR+ (Experiment 2)	Asian	Caucasian	Caucasian	Asian
AF/CUF: AR+ (Experiment 2)	Asian	Caucasian	Asian	Caucasian

The infant was seated in the parent’s lap roughly 60 cm from the display table. The parent sat in a rolling chair that allowed the parent to change location or orientation when instructed by the experimenter. Before each distribution episode began, the parent was instructed to turn to orient away from the table so that neither the parent nor the infant could see the display. The parent was also instructed to gaze neutrally at the top of the infant’s head and to avoid interacting with their infant during the procedure.

During the distribution, the infant watched a total of four distribution episodes: two that resulted in fair outcomes (a 2:2 distribution of toys) and two that resulted in unfair outcomes (a 1:3 distribution of toys). The distribution outcomes alternated, and each distributor consistently allocated toys either fairly (2:2), or unfairly (1:3). The recipient of the lesser distribution for the unfair outcome was consistent across both episodes. The distribution episodes were conducted such that each distributor was unaware of whether she was the fair or unfair distributor to avoid experimental bias. This feature of the distribution phase is a critical part of the procedure because if distributors are aware of the outcomes of their actions, this awareness may (inadvertently or unconsciously) influence their behavior on the test trials in subtle ways that can be hard to detect (e.g., the fair actor being slightly more positive toward the infant, etc.), which may artifactually create the experimental effect. Our procedure allows us to bypass this possibility.

To begin the distribution, the experimenter instructed the parent to turn to face the display table. One distributor and two recipients were seated at the table. The distributor knelt behind the table such that the table occluded any actions below her waist. Recipients were seated on either side of the distributor. All actors gazed neutrally down until the procedure began. After a 3-s delay (to ensure the infant was attending to the display table), a distribution episode began.

In the greeting phase, the distributor greeted the infant by saying, “Hello.” Next, the distributor greeted the recipient to her left, saying “Hi” and the recipient looked up and said “Hello.” The distributor and the recipient to her right repeated this procedure.

**FIGURE 1:**
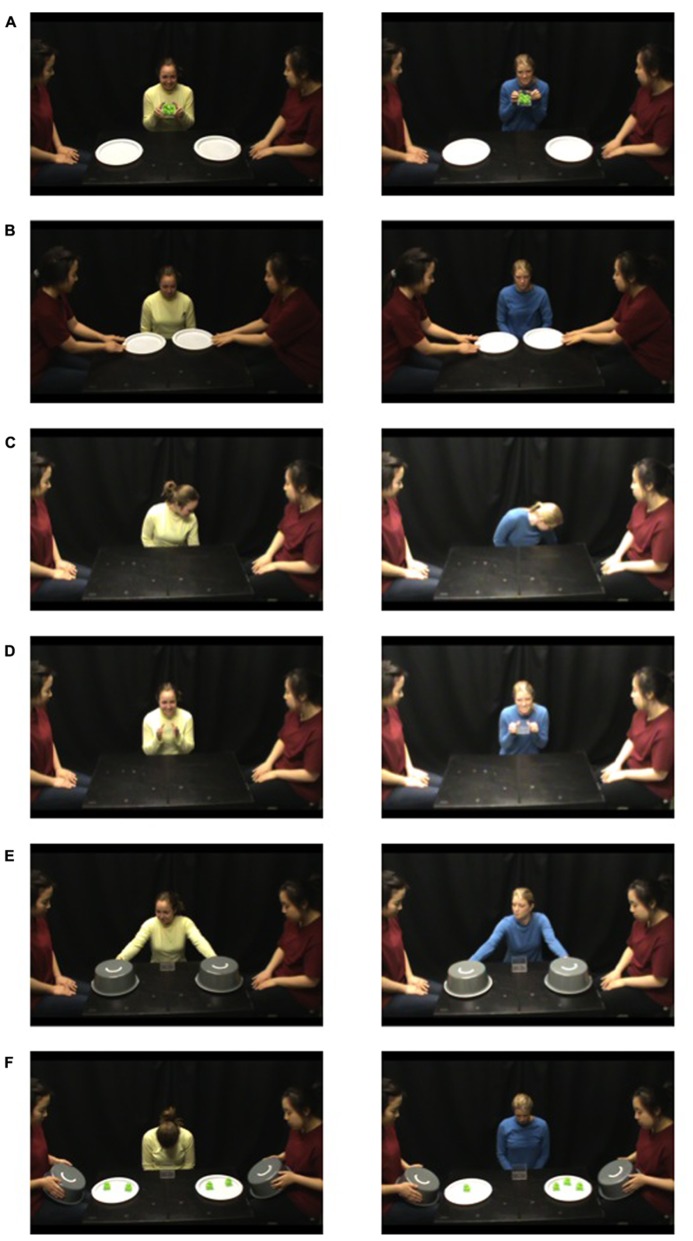
**Fair distribution episode (see left column) and unfair distribution episode (see right column). (A)** The distributor lifted up a transparent bin containing four toys and said “Wow!” **(B)** Both recipients simultaneously said “Please” and pushed the two containers toward the distributor. **(C)** The distributor pretended to distribute toys into the container on her left side (pictured), and then the container on her right side (not pictured). **(D)** The distributor held up the empty transparent bin and said “All gone!” **(E)** The distributor gave one container to each recipient saying “here.” **(F)** The distributor looked down with her eyes closed, and the recipients simultaneously lifted the lids to reveal the number of toys they received.

In the distribution phase, the distributor lifted up a transparent bin containing four toys and said “Wow” (see **Figure 1A**). Both recipients simultaneously said “Please” and pushed the two containers toward the distributor (**Figure 1B**). The distributor took the containers, simultaneously placed them on the floor behind the table, and appeared to distribute toys into each container; the containers were occluded from the infant’s view by the table (**Figure 1C**). The distributor then held up the now-empty transparent bin, said “All gone” (**Figure 1D**) and placed the transparent bin on the table. Next, the distributor lifted identical opaque lids within the infant’s view and simultaneously lowered them, pretending to cover the containers. In reality, in order to ensure that distributors were unaware of whether they were acting fairly or unfairly, distributors did not actually distribute the toys; an identical set of containers had been pre-prepared with toy allocations, covered with opaque lids, and hidden behind the table. Then, the distributor lifted the pre-prepared covered containers so they were in view of the infant, gave one container to each recipient saying “here” (**Figure 1E**), and looked down with her eyes closed (so she remained unaware of the outcome).

In the outcome phase, the recipients simultaneously lifted the lids to reveal the number of toys they received and the infant viewed the static allocation outcome for 20 s (**Figure 1F**). Then, the experimenter asked the parent to turn so that the infant was no longer facing the display and the actors reset the display.

The infant watched a total of four distribution episodes. Each distributor distributed a set of green plastic frogs (episodes 1 and 2), and then a set of yellow Lego bricks (episodes 3 and 4). The outcomes (2:2 versus 1:3) alternated each episode. The first outcome (2:2 versus 1:3), and the side of the advantaged recipient (left versus right) were counterbalanced across infants. Throughout the procedure, all actors’ actions were timed to a metronome to ensure consistency of timing across episodes and across the different distributors. The total duration of each distribution episode was 85 s.

***Choice trials*** Following the distribution phase, the infant received three trials in which she could choose between the fair and unfair distributors. The side of the fair distributor (left or right) was counterbalanced.

During Trial 1, the infant was seated in the parent’s lap facing away from the table, at a marked centered location 60 cm from the display table. The parent was instructed to hold the infant firmly by the waist to keep her in position in the middle of the parent’s lap. The fair and unfair distributors sat at the table equidistant from the infant. To begin the trial, the experimenter asked the parent to turn to face the display table. After a 3-s delay (to ensure the infant had adequate time to encode the distributors and their respective locations), the distributors simultaneously smiled and made eye contact with the infant, and then simultaneously extended identical octopus bath toys to the infant at marked locations 80 cm apart at the edge of the table. After a 3-s delay (to allow the infant to encode the toys), the experimenter instructed the parent to move up to the edge of the table with the infant centered between the distributors. During this response period, the distributors maintained eye contact and smiled at the infant in a static position with their arms extended. The trial ended after the infant took a toy or after 30 s elapsed. Once the trial ended, the experimenter asked the parent to move back to their original position facing away from the display table. If the infant had selected a toy, the experimenter retrieved it.

Trial 2 was identical to Trial 1, except before offering the toys to the infant, the distributors simultaneously said, “Wow.” Then the distributors placed the toys on the table 80 cm apart, and looked down. The trial ended when the infant took a toy, or after 30 s elapsed.

Before Trial 3 began, the parent and infant faced the table. Distributors moved behind the parent to sit on the floor at marked locations 2.13 m apart on opposite sides of the room and began stacking blocks. Next, the experimenter instructed the parent to turn to face the wall and the infant watched the distributors stack blocks in an identical manner for 20 s. The experimenter then instructed the parent to place the infant on a mark equidistant from the two distributors and release the infant so she was allowed to move freely. The trial ended when the infant approached a distributor and interacted with the blocks, or after 45 s elapsed. The choice was operationalized as the actor toward whom the participant moved nearest, as coded by an observer blind to condition and hypotheses from a video angle perpendicular to the two actors. Infants had to take at least one step toward an actor to be coded as an approach.

Our motivation for including three different test trial types was threefold. First, we wanted to investigate infants’ social selections and affiliative patterns generally. We thus sought to feature multiple test trials that measure the same underlying construct but that differ in their surface features to better position us to draw conclusions about infants’ social selections more broadly, versus their performance on one particular trial type. Second, based on pilot work we found that by varying the surface features of the test trials we could increase both the number of trials we could administer as well as the number of trials that infants made a choice on, by decreasing boredom, inattentiveness and fussiness. Finally, the use of three unique trials allowed us to strike a balance between (a) the task’s resemblance to a true social interaction, (b) the likelihood that the task would induce stranger anxiety, and (c) standardizing actors’ interactions with the infant. Trials 1 and 2 require the infant select a toy offered by an actor and were designed to minimize stranger anxiety, while Trial 3 requires that the infant approach an actor and may be a more direct selection of a social partner. Using toys rather than a direct interaction also helped to achieve this balance, allowing tasks that were sufficiently social while reducing stranger anxiety; pilot work revealed that tasks in which infants select toys offered by experimenters reduced indices of stranger anxiety than tasks in which infants interacted with the experimenters directly. Another benefit of using toys is that it eliminated actors’ contingent responding to the infants, ensuring the different actors’ interactions with the infant were identical within each testing session and across different testing sessions.

***Coding and reliability***. Infants’ distributor selections (fair versus unfair) were coded by a coder unaware of the respective roles of the distributors. For Trials 1 and 2, infants’ choices were recorded as the distributor from whom the infant first selected the toy. For Trial 3, infants’ choices were recorded as the distributor whom infants approached. On the rare occasion the infants approached both distributors (*n* = 3), infants’ choices were recorded as the distributor to whom the infant got closest.

A secondary coder, unaware of the distributors respective roles and also unaware of the primary coder’s responses, coded 25% of the sample to establish inter-observer reliability. Coders agreed on infants’ choices on 100% of trials.

### Results And Discussion

#### Infants’ selection of the fair distributor

A fair choice score was calculated by dividing the number of fair distributor choices by the total number of choices in the three choice trials. Participants were only included in the overall fair choice score if they made choices on at least two of the three trials; *n* = 1 infants in the CF/CUF condition and *n* = 3 in the AF/CUF condition were dropped for failing to meet this criteria. Given that we had a directional prediction that infants’ scores would be significantly *above* chance, one-tailed *p*-values are reported.

**FIGURE 2:**
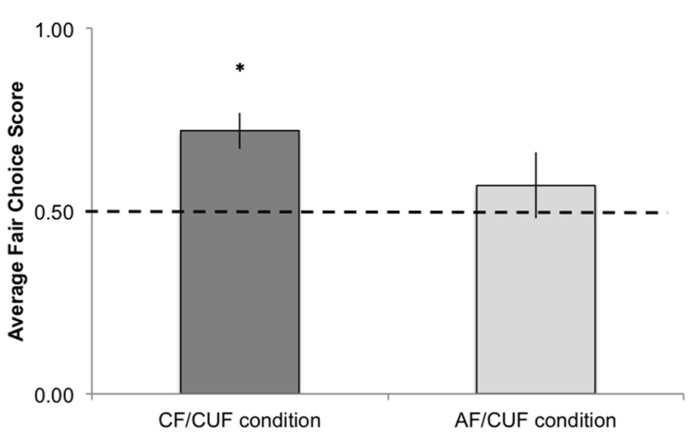
**Average fair choice scores in the CF/CUF and AF/CUF conditions.** *p < 0.05.

We first investigated whether infants’ overall fair choice score differed as a function of condition: there was a marginally significant difference in infants’ selection of the fair distributor in the CF/CUF condition (Caucasian Fair / Caucasian Unfair, *M* = 0.72, SE = 0.05) versus the AF/CUF condition (Asian Fair / Caucasian Unfair, *M* = 0.57, SE = 0.09), *t*(34) = 1.50, *p* = 0.07, *d* = 0.51. A one-sample t-test revealed that infants in the CF/CUF condition selected the fair actor at rates significantly above chance (where chance = 0.50): *t*(18) = 4.05, *p *= 0.0005, *d* = 1.91 (**Figure 2**). However, in the AF/CUF condition, infants’ fair choice scores did not differ significantly from chance, *t*(16) = 0.79, *p *= 0.22,* d* = 0.40.

To further investigate infants’ fair actor selections within each condition we conducted binomial tests on each of the test trials. In the CF/CUF condition, infants selected the fair actor at rates above chance on two of the three trials: 77% of infants selected the fair actor on Trial 1 (*p* = 0.045), 63% of infants selected the fair actor on Trial 2 (*p* = 0.18), and 75% of infants selected the fair actor on Trial 3 (*p* = 0.039). In contrast, infants in the AF/CUF condition selected the fair actor at rates that did not differ from chance on Trials 1 and 3 (58% selected the fair actor on Trial 1, *p* = 0.385; 50% of infants selected the fair actor on Trial 3, *p* = 1.0) and differed from chance marginally on Trial 2 (68% of infants selected the fair actor on Trial 2, *p* = 0.084)^3^.

The results of Experiment 1 suggest that infants select distributors on the basis of prior fair (versus unfair) behavior when the race of the distributors is held constant, suggesting that infants prefer to interact with others who abide by fairness norms, at least under certain circumstances. Thus, our findings suggest that by 15 months of age, infants’ fairness concerns guide not only their visual responses but also their social selections. Importantly, infants’ preference for the fair actor was present under conditions in which the actors were acting toward recipients of a different race. As some scholars have suggested that moral norms are those that apply universally to members of all social categories, it may be the case that infants are thus treating fairness violations as moral transgressions, rather than social conventional violations (Turiel, 1983). For example, recent work suggests young children implicitly draw this distinction between moral and conventional norms; three-year-old children appear to believe that moral norms apply in interactions with both ingroup and outgroup members, while conventional norms are uniquely restricted to interactions with ingroup members (Schmidt et al., 2012). Our findings that infants appear to recognize fairness norms apply in interactions with outgroup recipients raises the possibility that infants are similarly sensitive to the moral, as opposed to conventional, basis of fairness norms.

Our findings also suggest that when fairness and race are pitted against one another (e.g., fair Asian actor versus unfair Caucasian actor), there is no evidence that infants systematically select the fair actor at rates above chance. These findings suggest that infants may attempt to integrate both social category information, operationalized here as race, and social history, operationalized here as prior fair and unfair behavior, in making their social selections and that these factors compete with one another in infants’ social decision making.

## EXPERIMENT 2

The results of Experiment 1 established that infants systematically select fair distributors when race is kept constant. However, when race is pitted against fair behavior infants do not systematically select the fair actor. Experiment 2 asked whether, in addition to considering race and fairness in their social selections, infants also consider the racial identity of the advantaged individual.

Infants were tested in either a Caucasian Fair/Asian Unfair condition, or an Asian Fair/Caucasian Unfair condition. In both conditions the recipients consisted of an Asian individual and a Caucasian individual. For half of the infants in each condition the Asian individual was advantaged by the unfair actor (e.g., received more toys); for the remaining half the Caucasian recipient was advantaged by the unfair actor. Given the results of Experiment 1, suggesting that infants consider both race and fairness in their selections, we thought it was unlikely that infants would select actors solely on the basis of prior fair behavior (e.g., systematically picking the fair actor), or solely on the basis of race (e.g., systematically selecting the Caucasian actor). Instead, we predicted one of two patterns of results.

The first possibility is that infants may select the fair actor over the unfair actor when the fair actor is Caucasian (and the unfair actor is Asian), but not when the fair actor is Asian (and the unfair actor is Caucasian), ignoring the consequences of the fair and unfair actors’ behavior for the recipients. An alternative possibility is that infants may consider the consequences of the distributors’ actions for the recipients as a function of the recipients’ racial identities. For half of the infants in Experiment 2, the unfair actor advantaged the Asian recipient (over the Caucasian recipient), and for half of the infants, unfair actor advantaged the Caucasian recipient (over the Asian recipient). If infants are sensitive to the consequences of the distributor’s actions for the recipient as a function of the recipients’ racial identities, then infants’ social selections may vary as a function of who the *unfair* actor advantages. Specifically, we hypothesized that Caucasian infants would be more likely to select the fair actor when the unfair actor advantaged the recipient that was of a different race than the infant (i.e., the Asian recipient), than when she advantaged a recipient that was of the same race as the infant (i.e., the Caucasian recipient).

### METHODS

#### Participants

Forty 15-month-old Caucasian infants took part in Experiment 2 (19 females; mean age = 15 months, 10 days, range = 14 months, 28 days to 15 months, 27 days). Infants were randomly assigned to the Caucasian Fair/Asian Unfair condition (henceforth CF/AUF; *n* = 20, 10 females, mean age = 15 months, 10 days) or the Asian Fair/Caucasian Unfair condition (henceforth AF/CUF; *n* = 20, 9 females, mean age = 15 months, 11 days). All infants were full-term and typically developing. Participants were recruited from a database of parents who had volunteered to participate in experimental studies.

Data from 10 additional infants were excluded due to failure to respond at all during the choice trials (*n = *3 in the CF/AUF condition, *n* = 1 in the AF/CUF), fussiness (*n* = 2 in the CF/AUF condition, *n* = 1 in the AF/CUF) or procedural errors (*n* = 2 in the CF/AUF condition), or due to parental influence (*n* = 1 in the AF/CUF condition).

#### Procedure

Experiment 2 was identical to Experiment 1 except for the race of the various actors. For both conditions, one distributor was Caucasian and the other distributor was Asian, and one recipient was Caucasian and the other recipient was Asian. For each condition, the race of the recipient who received three toys from the unfair distributor (henceforth, the “advantaged recipient”) was counterbalanced; for half of the infants in each condition the advantaged recipient was Caucasian, and for half of the infants in each condition the advantaged recipient was Asian.

#### Coding and reliability

Infants’ choices were coded as in Experiment 1. As in Experiment 1, when infants approached both distributors (*n* = 3), infants’ choices were recorded as the distributor to whom the infant got closest. A second coder coded 25% of the sample to establish inter-observer reliability. Coders agreed on the infants’ choices on 100% of the trials.

### RESULTS AND DISCUSSION

#### Infants’ selection of the fair distributor

As in Experiment 1, a fair choice score was calculated by dividing the number of fair distributor choices by the total number of choices in the three choice trials. As in Experiment 1, participants were only included if they made choices on at least two of the three trials; *n* = 3 infants in the CF/AUF condition, *n* = 3 in the AF/CUF condition were dropped for failing to meet this criteria. Fair choice scores did not significantly differ between the CF/AUF condition (Caucasian Fair/Asian Unfair, *M* = 0.45, SE = 0.07) and the AF/CUF condition (Asian Fair/Caucasian Unfair, *M* = 0.55, SE = 0.07), *t*(32) = 1.06, *p* = 0.15, *d* = 0.37. Similarly, fair choice scores did not significantly differ from chance in either the CF/AUF condition: *t*(16) = 0.75, *p* = 0.23, *d* = 0.38, or the AF/CUF condition: *t*(16) = 0.75, *p* = 0.23,* d* = 0.38.

#### Infants’ selection of the fair distributor as a function of the race of the recipient advantaged by the unfair distributor

**FIGURE 3:**
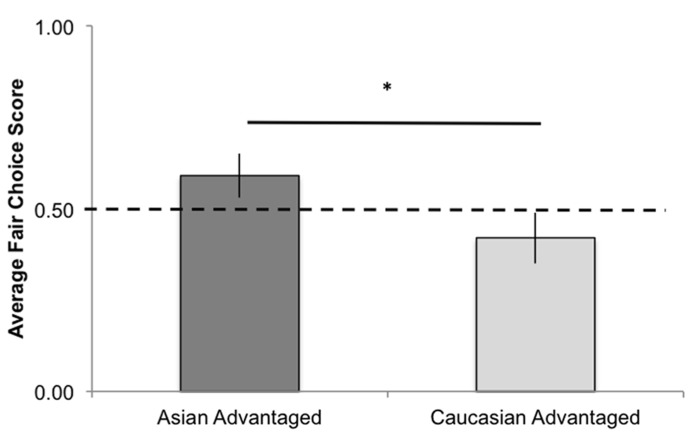
**Average fair choice scores in Experiment 2 as a function of the race of the recipient advantaged by the unfair actor.** *p < 0.05.

We then investigated whether infants’ choices were influenced by the race of the advantaged recipient (i.e., the recipient who receives three toys from the unfair distributor). Overall, there was a significant effect of the recipient who was advantaged (i.e. Asian versus Caucasian) on infants’ selections of the fair distributor, *t*(32) = 2.00, *p *= 0.03,* d* = 0.71. Infants were more likely to select the fair actor when the unfair actor advantaged the Asian recipient, *M* = 0.59, SE = 0.06, than when the unfair actor advantaged the Caucasian recipient,* M *= 0.42, SE = 0.07 (**Figure 3**). A one-sample *t*-test revealed infants’ selections of the fair actor was marginally above chance (where chance = 0.50) when the unfair actor advantaged the Asian recipient: *t*(15) = 1.65, *p* = 0.06. Infants’ selections of the fair actor were not significantly different from chance when the unfair actor advantaged the Caucasian recipient, *t*(17) = 1.26, *p* = 0.13. Performance on individual test trials was consistent with this pattern of findings (although binomial tests on each trial were not significant, *p*s > 0.05, presumably due to lack of power). When the unfair actor advantaged the Asian recipient, 73% of infants selected the fair actor on Trial 1, 58% of infants selected the fair actor on Trial 2, and 57% of infants selected the fair actor on Trial 3. In contrast, when the unfair actor advantaged the Caucasian recipient, 38% of infants selected the fair actor on Trial 1, 35% of infants selected the fair actor on Trial 2, and 47% of infants selected the fair actor on Trial 3.

The results of the second experiment suggest infants were no more likely to select the fair distributor than the unfair distributor when the recipients belonged to different racial categories. However, infants’ selections varied systematically as a function of the race of the advantaged recipient; infants were more likely to select the fair distributor when the unfair distributor advantaged the Asian recipient. These findings suggest that infants may also make social selections based on the consequences that a given individual’s behavior may have for individuals that are of the same versus a different race as infants.

## GENERAL DISCUSSION

Our findings provide evidence that infants select social partners on the basis of their prior fair versus unfair behavior. Past work suggests infants expect individuals to distribute goods fairly to recipients. Our findings build on this work by showing that infants’ fairness concerns also actively guide their social preferences and social selections. Critically, infants chose to interact with the fair actor even when both recipients could be construed as belonging to a different social category than both the recipients and the infants. Thus, infants appear to apply fairness norms when the victim of a fairness violation is of a different social category. Because a moral norm is often defined as one that is universally applied, this may provide initial evidence that infants construe fairness as a moral norm, rather than a social convention (Turiel, 1983). However, to fully draw this conclusion, future work would need to address whether infants expect outgroup members to abide by fairness norms. Future work similar to the current study with two Asian distributors (e.g., an Asian Fair/Asian Unfair condition) could provide evidence to support this conclusion.

An outstanding question concerns whether infants’ social selections reflect the formation of overarching evaluations: forming a positive evaluation of individuals who behave fairly and a negative evaluation of individuals who behave unfairly, and using these evaluations to guide their social selections. Alternatively, infants’ choices may merely reflect self-interested concerns about the possible consequences of future interactions between the infant and potential social partners. Future work may disentangle these possibilities.

Another open question is how infants’ awareness of such norms differs from that of older children. Our findings suggest that infants have an emerging understanding of fairness norms that operate according to (at least) some of the same moral principles as adults and older children. However, we assume infants’ awareness of such norms is primarily implicit. A critical question for future work concerns how children develop an explicit awareness of fairness norms and other socio-moral considerations.

The findings of Experiment 1 also indicated, however, that when distributor race is pitted against prior fair behavior, infants do not systematically select the fair distributor. These findings suggest infants attempt to incorporate information about the individuals’ races when making social selections, and may weigh race and fairness as competing dimensions in their social selections. An alternative explanation for the findings of Experiment 1 is that infants may struggle to incorporate multiple social dimensions when making social selections, such that when two or more dimensions are present, infants select social partners at chance. However, this explanation is ruled out by the findings of Experiment 2, in which fairness was crossed with both the race of the distributor *and* the race of the recipient and infants selected social partners systematically based on who (i.e., which race recipient) the unfair distributor advantaged.

Experiment 2 demonstrated that infants were more likely to select the fair actor when the unfair actor advantaged an Asian individual than when she advantaged a Caucasian individual. Given that all of the infants in the sample were Caucasian, these findings may suggest that over and above whether an actor behaves fairly, infants may focus on the consequences of the distribution for members of their own race and its implications for their own future interactions with the distributors, as it may be advantageous to interact with a person who shows preferential treatment to the infant’s own race. Infants may prefer the fair distributor in this situation either because the unfair distributor (a) *advantaged* the Asian recipient, or (b) *disadvantaged* the Caucasian recipient. Because the unfair distributor both gave more goods to the Asian recipient than did the fair distributor, and fewer goods to the Caucasian recipient than did the fair distributor, the current study does not address this question. However, previous studies suggest infants’ preferences may be based on both of these aspects; 14-month-old infants prefer agents who help a puppet that is similar to the infant (e.g., prefers the same kind of food), but prefer agents who harm a puppet that is dissimilar (e.g., prefers different foods) (Hamlin et al., 2013). Future studies may cleave apart these two possibilities by introducing one distributor who gives more to the Asian recipient (but gives the same amount to the Caucasian recipient as does the fair distributor), and one who disadvantages the Caucasian recipient (but gives the same amount to the Asian recipient as does the fair distributor).

An additional question concerns whether infants are making their social selections based on shared category membership with the agents involved in the display (e.g., based on the fact that the infant and agents are of the same race) or in terms of considerations for Caucasian recipients *per se*. Because all of the participants in the current study were Caucasian, the current study cannot distinguish between these possibilities. If infants’ choices are driven by shared category membership, future studies using the same design as Experiment 2 with Asian participants should yield an opposite pattern of findings (e.g., selections of the fair actor should be higher when the unfair actor advantages the Caucasian recipient). This is likely the case, given that Asian and Caucasian infants have shown opposite looking time patterns in investigations of infants’ perception of Asian versus Caucasian faces, i.e., Asian infants look longer at Asian faces and Caucasian infants look longer at Caucasian faces (Kelly et al., 2007).

A related question concerns whether infants’ fairness concerns guide their selection of social partners when both the fair and unfair individuals are of another race, e.g., if Caucasian infants were given the option between fair and unfair actors who are both Asian. Our work suggests that Caucasian infants do prefer fair actors over unfair actors when they are both Caucasian, so it is reasonable to expect this would be the case when they are both Asian. An alternative possibility is that Caucasian infants do not have any expectations about how other-race actors should distribute goods. Given, however, that infants in Experiment 2 selectively chose fair actors when the unfair actor advantaged an Asian recipient, it suggests Caucasian infants may hold Asian actors to the same principles as Caucasian actors, and that one would see this same pattern if both fair and unfair actors were Asian.

The extent to which infants focus on the consequences of the distribution for members of their own race parallels similar findings with adults: although adults who had been divided into artificial groups (ostensibly based on perceptual processing styles) condemned ingroup favoritism, they tended to have an *implicit* preference for a person who showed ingroup favoritism over a person who was egalitarian (Castelli et al., 2008). Systematic biases for ingroup members do not appear to emerge until 3 or 4 years of age (Katz and Kofkin, 1997; Dunham et al., 2013); however, our findings may signal the onset of an emerging implicit awareness of social categories and the implications of these categories for people’s real-world behavior and consequences of this behavior.

Our findings are consistent with previous studies showing that race affects infants’ visual attention, leading to a preference for looking at own- versus other-race individuals (Kelly et al., 2005). However, our findings go beyond looking preferences, which may rely on lower-level processes, and suggest infants actively use this information to coordinate their social selections. The current findings are in contrast to some previous work on infants’ social preferences that finds that infants do not consider race when given the opportunities to select toys associated with White versus Blacks actors (Kinzler and Spelke, 2011). One possibility for this difference is that in our procedure infants observed and interacted with live (as opposed to televised) adults; thus, it is possible infants perceived our task as having realistic consequences for future interactions with these individuals. Another strength of the current study is that we utilized multiple exemplars of Caucasian and Asian individuals, in multiple different pairings, which de-conflates race and personal identity. Thus, our findings could not be accounted for by a preference for one a particular individual over another. Moreover, because infants were recruited from different locales across these studies, it is possible the extent of infants’ exposure to same- and other-race adults may have differed. One possibility is that infants in more racially diverse cities may have more exposure to other-race individuals and may be less likely to use race as a marker of social category membership.

In conclusion, the results of the current study suggest that infants can use fairness concerns to guide their social selections. However, infants also take into consideration the race of individuals, and the consequences of the behavior of these individuals for their own- versus other-race individuals. Indeed, the results of Experiment 2 suggest that when given the opportunity to select individuals on the basis of fairness, on the basis of race, or based on the consequences of the distributor’s actions for own- versus other-race individuals, infants most strongly consider the consequences for own- versus other-race members. These findings may suggest that when confronted with selecting between individuals on the basis of who abides by a fairness norm versus on the basis of who advantages own-race (versus other-race) individuals, infants may more strongly weight the consequences for individuals of their own race, and, by extension, for the self. Thus, infants may strategically select social partners who previously advantaged members of their own social category, suggesting that they may use group membership to predict the consequences of future interactions for themselves. Thus, our work is consistent with the conclusion that infants and young children may be strategic in their prosocial considerations (Dunfield and Kuhlmeier, 2010; Vaish et al., 2010; Shaw et al., 2012), factoring in not only whether an individual acts fairly, but also the potential consequences of this behavior for their own interactions with others.

## AUTHOR CONTRIBUTIONS

Monica P. Burns and Jessica A. Sommerville conceived and designed the experiments. Monica P. Burns performed the experiments. Monica P. Burns and Jessica A. Sommerville analyzed the data and wrote the paper.

## Conflict of Interest Statement

The authors declare that the research was conducted in the absence of any commercial or financial relationships that could be construed as a potential conflict of interest.

## Footnotes

1 These trials were embedded in a larger set of social tasks. Because response rates in other tasks were low (e.g., less than 25% of infants provided responses), we focused our analyses on these three trials.

2 Past studies have conflated individual identity with category membership by utilizing only one exemplar of the category and drawing inferences about the entire category (e.g., one black person and one white person, Kinzler and Spelke, 2011; one male and one female, Rind and Bordia, 1996). Thus, it may be the case that those findings represent preferences for particular individuals, rather than preferences for particular social groups. To avoid this conflation, and to ensure we were investigating the selection of individuals on the basis of engaging in past behaviors and on the basis of category membership, we used multiple Caucasian and Asian actors to play distributors and recipients across the two studies. Actors were randomly paired with one another, constrained by the race required for a given condition. Roughly half of the time a given actor acted as the fair (versus unfair) distributor. In Experiment 2, when a given actor was the unfair distributor, roughly half of the time she advantaged the Caucasian (versus Asian) recipient.

3 One potential reason that infants selections on Trial 2 did not differ from chance may be that Trial 1 demonstrates to the infant that, at least in the context of offering toys, both actors are equally likely to offer a toy to the infant and thus the infant chooses randomly on the second trial. An alternative possibility is that because trial 2 is similar to trial 1, infants may feel the actors are encouraging them to make a different choice.

## References

[B1] Aboud F. E. Mendelson M. J. Purdy K. T. (2003). Cross-race peer relations and friendship quality. *Int. J. Behav. Dev.* 27 165–173 10.1080/01650250244000164

[B2] Bellmore A. D. Nishina A. Witkow M. R. Graham S. Juvonen J. (2007). The influence of classroom ethnic composition on same- and other-ethnicity peer nominations in middle school. *Soc. Dev.* 16 720–740 10.1111/j.1467-9507.2007.00404.x

[B4] Castelli L. De Amicis L. Sherman S. J. (2007). The loyal member effect: on the preference for ingroup members who engage in exclusive relations with the ingroup. *Dev. Psychol.* 43 1347–1359 10.1037/0012-1649.43.6.1347 18020816

[B3] Castelli L. Tomelleri S. Zogmaister C. (2008). Implicit ingroup metafavoritism: subtle preference for ingroup members displaying ingroup bias. *Personal. Soc. Psychol. Bull.* 34 807–818 10.1177/0146167208315210 18359956

[B5] Dawes C. T. Fowler J. H. Johnson T. McElreath R. Smirnov O. (2007). Egalitarian motives in humans. *Nature* 446 794–796 10.1038/nature05651 17429399

[B6] Deutsch M. (1975). Equity, equality and need: what determines which value will be used as the basis of distributive justice? *J. Soc. Issues* 31 137–149 10.1111/j.1540-4560.1975.tb01000.x

[B7] Dunfield K. A. Kuhlmeier V. A. (2010). Intention-mediated selective helping in infancy. *Psychol. Sci.* 21 523–527 10.1177/0956797610364119 20424094

[B8] Dunfield K. A. Kuhlmeier V. A. Murphy L. (2013). Children’s use of communicative intent in the selection of cooperative partners. *PLoS ONE* 8:e61804 10.1371/journal.pone.0061804 PMC3633994 23626731

[B9] Dunham Y. Chen E. E. Banaji M. R. (2013). Two signatures of implicit intergroup attitudes: developmental invariance and early enculturation. *Psychol. Sci.* 24 860–868 10.1177/0956797612463081 23558550

[B10] Fehr E. Fischbacher U. (2003). The nature of human altruism. *Nature* 425 785–791 10.1038/nature02043 14574401

[B11] Fiske S. T. Neuberg S. L. (1990). “A continuum of impression formation, from category-based to individuating processes: influences of information and motivation on attention and interpretation,” in *Advances in Experimental Social Psychology* Vol. 23 ed. Zanna M. P. (Waterloo, ON: Academic Press, Inc.) 1–74

[B12] Geraci A. Surian L. (2011). The developmental roots of fairness: infants’ reactions to equal and unequal distributions of resources. *Dev. Sci.* 14 1012–1020 10.1111/j.1467-7687.2011.01048.x 21884317

[B13] Hamlin J. Mahajan N. Liberman Z. Wynn K. (2013). Not like me = bad: infants prefer those who harm dissimilar others. *Psychol. Sci.* 24 589–594 10.1177/0956797612457785 23459869 PMC4374623

[B14] Hamlin J. K. Wynn K. (2011). Young infants prefer prosocial to antisocial others. *Cogn. Dev.* 26 30–39 10.1016/j.cogdev.2010.09.001 21499550 PMC3076932

[B15] Henrich J. Boyd R. Bowles S. Gintis H. Fehr E. Camerer C. (2005). Economic man in cross-cultural perspective: behavioral experiments in 15 small-scale societies. *Behav. Brain Sci.* 28 795–815 10.1017/S0140525X05000142 16372952

[B16] Hewstone M. Hantzi A. Johnston L. (1991). Social categorization and person memory: the pervasiveness of race as an organizing principle. *Eur. J. Soc. Psychol.* 21 517–528 10.1002/ejsp.2420210606

[B17] Johnson T. Dawes C. Fowler J. McElreath R. Smirnov O. (2009). The role of egalitarian motives in altruistic punishment. *Econ. Lett.* 102 192–194 10.1016/j.econlet.2009.01.003

[B18] Katz P. A. (2003). Racists or tolerant multiculturalists? How do they begin? *Am. Psychol.* 58 897–909 10.1037/0003-066X.58.11.897b 14609382

[B19] Katz P. A. Kofkin J. A. (1997). “Race, gender, and young children,” in *Developmental Psychopathology: Perspectives on Adjustment, Risk, and Disorder* eds Luthar S. S. Burack J. A. Cicchetti D. Weisz J. (New York: Cambridge University Press) 51–74

[B20] Kelly D. J. Liu S. Ge L. Quinn P. C. Slater A. M. Lee K. (2007). Cross-race preferences for same-race faces extend beyond the African versus Caucasian contrast in 3-month-old infants. *Infancy* 11 87–95 10.1207/s15327078in1101_4 18974853 PMC2575407

[B21] Kelly D. J. Quinn P. C. Slater A. M. Lee K. Gibson A. Smith M. (2005). Three-month-olds, but not newborns, prefer own-race faces. *Dev. Sci.* 8 F31–F36 10.1111/j.1467-7687.2005.0434a.x 16246233 PMC2566511

[B22] Kinzler K. D. Dupoux E. Spelke E. S. (2012). “Native” objects and collaborators: infants’ object choices and acts of giving reflect favor for native over foreign speakers. *J. Cogn. Dev.* 13 67–81 10.1080/15248372.2011.567200 23105918 PMC3478775

[B23] Kinzler K. D. Spelke E. S. (2011). Do infants show social preferences for people differing in race? *Cognition* 119 1–9 10.1016/j.cognition.2010.10.019 21334605 PMC3081609

[B24] Martin C. L. Fabes R. A. (2001). The stability and consequences of young children’s same-sex peer interactions. *Dev. Psychol.* 37 431–446 10.1037/0012-1649.37.3.431 11370917

[B25] McPherson M. Smith-Lovin L. Cook J. M. (2001). Birds of a feather: homophily in social networks. *Annu. Rev. Soc.* 27 415–444 10.1146/annurev.soc.27.1.415

[B26] Page T. Putterman L. Unel B. (2005). Voluntary association in public goods experiments: reciprocity, mimicry and efficiency. *Econ. J.* 115 1032–1053 10.1111/j.1468-0297.2005.01031.x

[B27] Platow M. J. Hoar S. Reid S. Harley K. Morrison D. (1997). Endorsement of distributively fair and unfair leaders in interpersonal and intergroup situations. *Eur. J. Soc. Psychol.* 27 465–494 10.1002/(SICI)1099-0992(199707)27:4<465::AID-EJSP817>3.0.CO;2-8

[B28] Rind B. Bordia P. (1996). Effect of restaurant tipping of male and female servers drawing a happy, smiling face on the backs of customers’ checks. *J. Appl. Soc. Psychol.* 26 218–225 10.1111/j.1559-1816.1996.tb01847.x

[B29] Schmidt M. F. Sommerville J. A. (2011). Fairness expectations and altruistic sharing in 15-month-old human infants. *PLoS ONE* 6:e23223 10.1371/journal.pone.0023223 PMC3188955 22003380

[B30] Schmidt M. F. H. Rakoczy H. Tomasello M. (2012). Young children enforce social norms selectively depending on the violator’s group affiliation. *Cognition* 124 325–333 10.1016/j.cognition.2012.06.004 22766522

[B31] Shaw A. DeScioli P. Olson K. R. (2012). Fairness versus favoritism in children. *Evol. Hum. Behav.* 33 736–745 10.1016/j.evolhumbehav.2012.06.001

[B32] Shin S. Burns M. P. Yun J. Sommerville J. A. (2011). When a friend doesn’t look like me: 12-month-old infants overcome race bias and prefer social individuals. *Poster Presented at the Biennial Meeting of the Society for Research and Child Development* Montreal

[B33] Shutts K. Roben C. K. P. Spelke E. S. (2013). Children’s use of social categories in thinking about people and social relationships. *J. Cogn. Dev.* 14 35–62 10.1080/15248372.2011.638686 23646000 PMC3640585

[B34] Sloane S. Baillargeon R. Premack D. (2012). Do infants have a sense of fairness? *Psychol. Sci.* 23 196–204 10.1177/0956797611422072 22258431 PMC3357325

[B35] Sommerville J. A. Schmidt M. F. H. Yun J. Burns M. (2013). The development of fairness expectations and prosocial behavior in the second year of life. *Infancy* 18 40–66 10.1111/j.1532-7078.2012.00129.x

[B36] Stangor C. Lynch L. Duan C. Glass B. (1992). Categorization of individuals on the basis of multiple social features. *J. Pers. Soc. Psychol.* 62 207–218 10.1037/0022-3514.62.2.207

[B37] Turiel E. (1983). *The Development of Social Knowledge: Morality and Convention*. Cambridge, MA: Cambridge University Press

[B38] Vaish A. Carpenter M. Tomasello M. (2010). Young children selectively avoid helping people with harmful intentions. *Child Dev.* 81 1661–1669 10.1111/j.1467-8624.2010.01500.x 21077854

